# Supplementary material for: ANKS1B in the Nucleus Accumbens Controls Escalated Cocaine Self‐Administration via Regulating CBP‐FoxO3 Complex
Source: Adv Sci (Weinh). 2026 Jun 2:e22949. Online ahead of print. doi: 10.1002/advs.202522949 (PMC13337124; doi:10.1002/advs.202522949)
Supplement: Supplementary file 1 — Supporting File 1: advs75935‐sup‐0001‐SuppMat.docx. [file ADVS-9999-e22949-s002.docx]

Supporting Information

**ANKS1B in the Nucleus Accumbens controls escalated cocaine self-administration via regulating CBP-FoxO3 complex**

Liping Yang,^1,2^ Xiaoxuan Wu,^1,2^ Xuan Chen^3^, Chao Peng^4^, Zihang Li^5^, Shumin Gao^1,2^, Shiqiu Meng^1,2^, Jing Dong^6^, Dong Wu^7^, Liying, Lv^8^, Ying Han^1,2^, Yanxue Xue^1,2^, Lin Lu^2,3,9^*, Jie Shi^1,2*^, Jianfeng Liu^6^*, Yan Sun^1,2^*


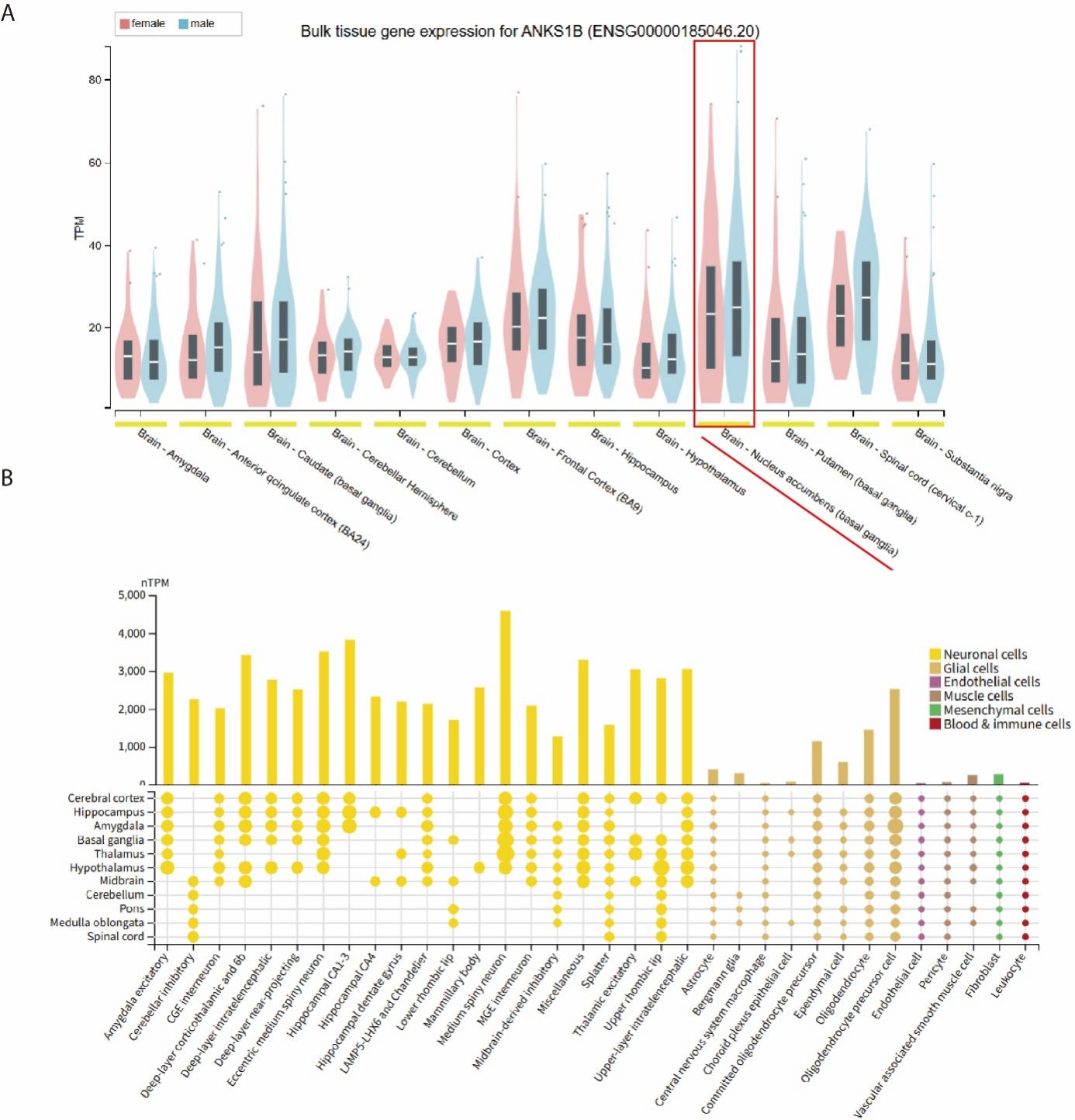


**Figure S1. Expression profile of *ANKS1B* in the brain.** **(A)** Violin plot showing the gene expression level (TPM: Transcripts Per Million) of *ANKS1B* in different regions of the human brain from the GTEx database bulk RNA-sequencing data. The data indicate that *ANKS1B* is expressed across multiple brain regions, including the Nucleus Accumbens (NAc, highlighted by the red box). **(B)** Single-cell transcriptomics data showing *ANKS1B* expression (nTPM: normalized Transcripts Per Million) in various brain cell types. The bar plot on top shows that *ANKS1B* is highly expressed predominantly in neuronal cells (yellow). The dot plot below further illustrates its distribution among specific neuronal subtypes.


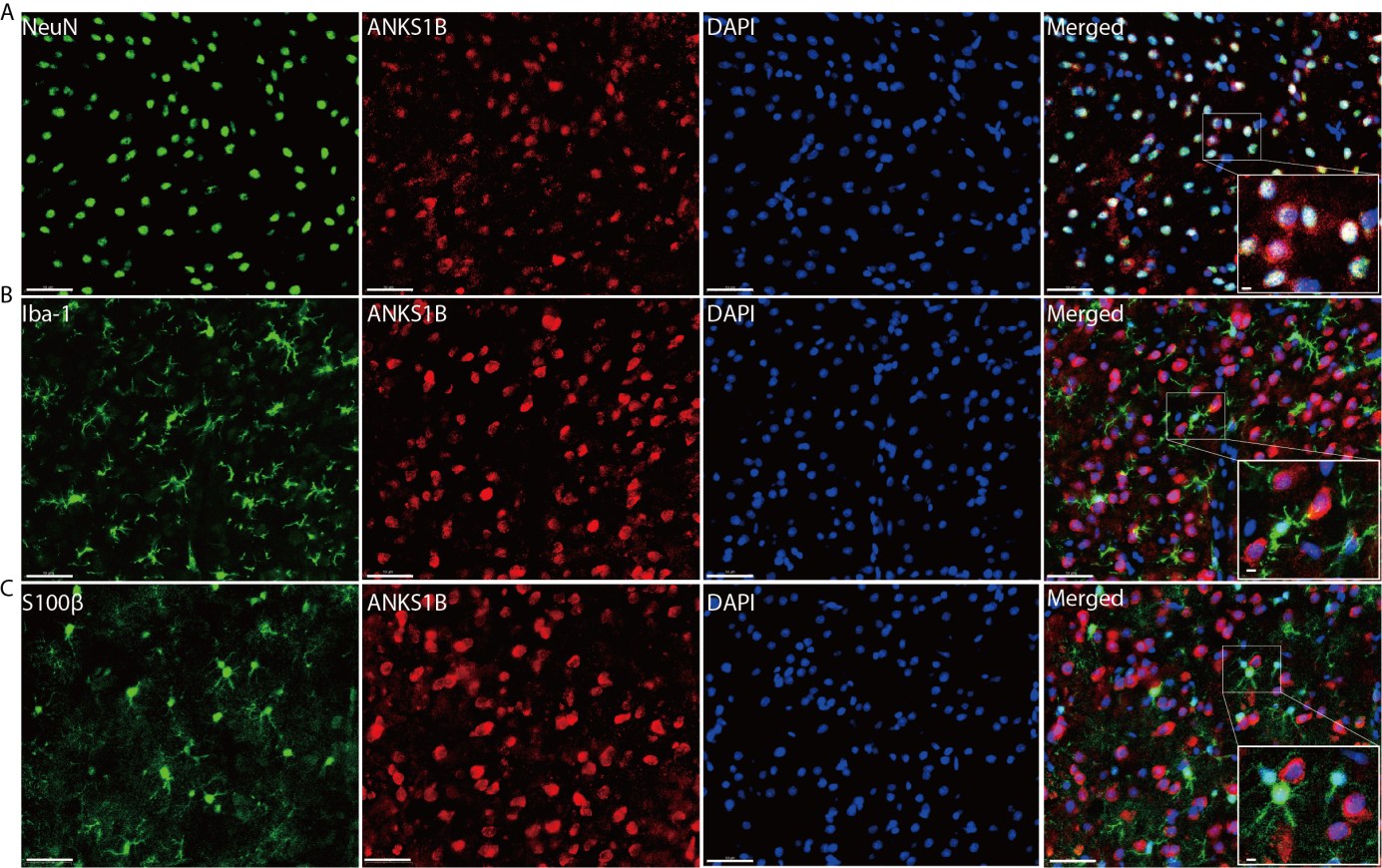


**Figure S2. ANKS1B is predominantly expressed in neurons within the NAc.** **(A)** Immunofluorescence co-staining shows extensive co-localization of ANKS1B (red) with the neuronal marker NeuN (green), indicating ANKS1B expression in neurons. **(B)** Co-staining of ANKS1B (red) with the microglial marker Iba-1 (green) shows no co-localization. **(C)** Co-staining of ANKS1B (red) with the astrocyte marker S100β (green) shows little to no co-localization. All scale bars, 50 µm and 5 µm.


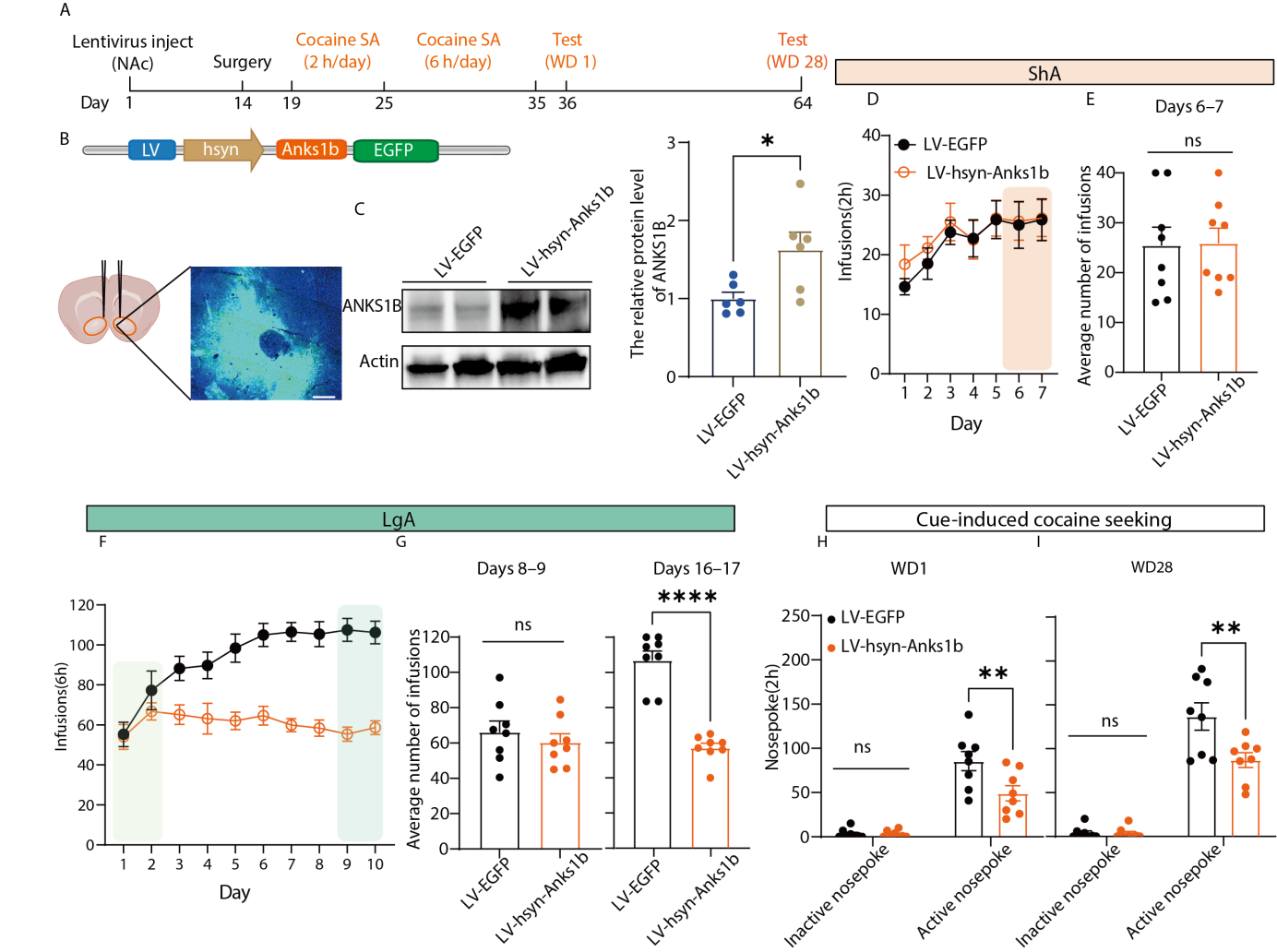


**Figure S3. ANKS1B overexpression in the NAc suppresses escalation of cocaine self-administration and cue-induced seeking. (A)** Experimental timeline showing lentivirus injection, cocaine self-administration, and WD testing. **(B)** Representative image showing LV-hsyn-ANKS1B-EGFP expression in the NAc. Scale bars, 100 μm. **(C)** Western blot validation confirming successful ANKS1B overexpression in the NAc compared with the control group (unpaired t-test, t_10_ = 2.66, P = 0.02, n = 6 per group). **(D)** ANKS1B overexpression did not alter cocaine acquisition during the ShA phase (n = 8 per group). **(E)** Comparison of cocaine infusions during the last 2 days of ShA (days 6–7) revealed no difference between groups (unpaired t-test, t_14_ = 0.09, P = 0.93). **(F)** ANKS1B overexpression prevented escalation of cocaine intake during the LgA phase. **(G)** Comparison of infusions on the first 2 days (days 8–9; unpaired t-test, t_14_ = 0.74, P = 0.47) and the last 2 days (days 16–17; t_14_ = 8.35, P < 0.01) of LgA. **(H and I)** Overexpression of ANKS1B significantly reduced cue-induced cocaine seeking (Active nosepoke) on both WD1 and WD28. (WD1: two-way RM ANOVA, genotype × nosepoke interactions: F_1, 14_ = 6.60, P = 0.02; post hoc, P = 0.01; WD28: two-way RM ANOVA, genotype × nosepoke interactions: F_1, 14_ = 6.64, P = 0.02; post hoc, P < 0.01; n = 8 per group). An EGFP-expressing lentiviral vector (LV-EGFP) was used as the control. Data are presented as mean ± SEM. P < 0.05 was considered statistically significant. Statistical significance was determined by two-way repeated-measures (RM) ANOVA followed by Bonferroni's multiple comparisons test.


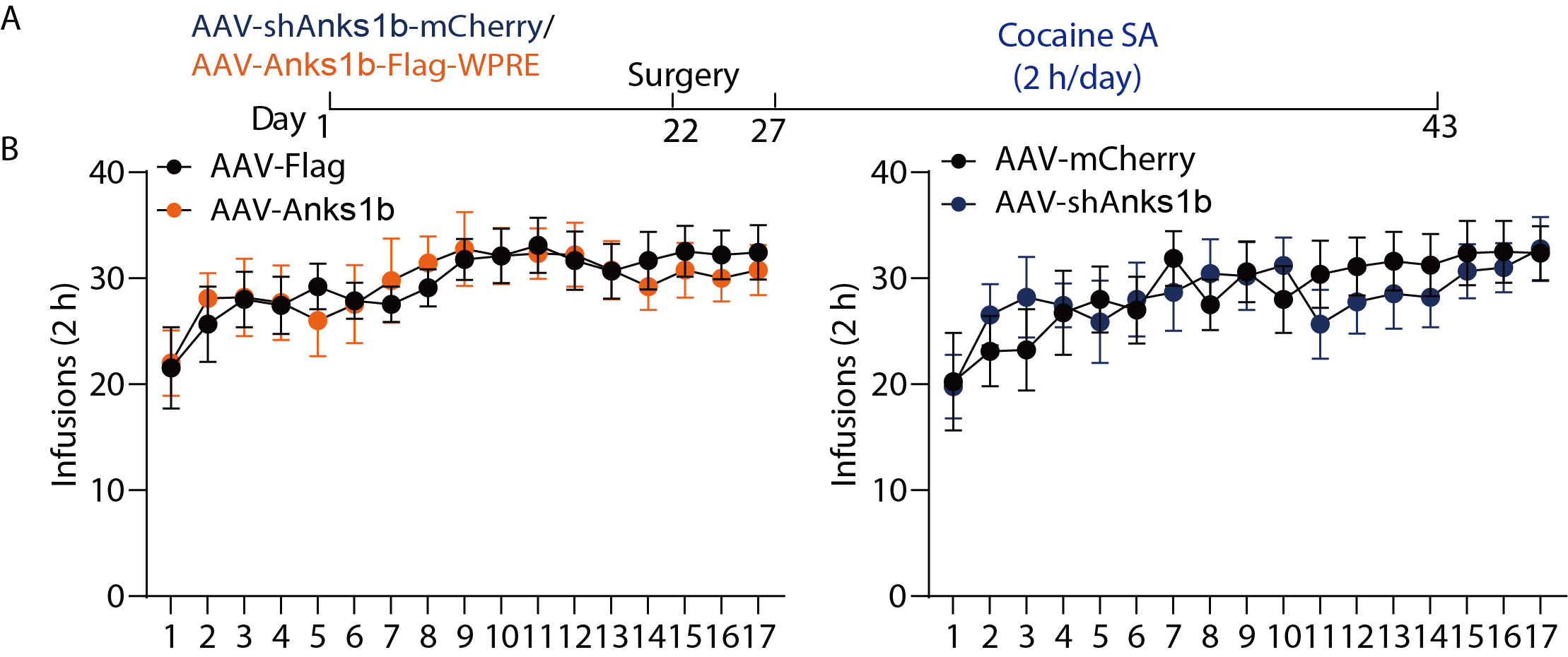


**Figure S4. Manipulation of ANKS1B levels in the NAc does not affect Short-access cocaine acquisition.** **(A)** Experimental timeline showing a 17-day acquisition period of cocaine self-administration (SA) under a short-access (2h/day) schedule. **(B)** Daily cocaine infusions. The left panel shows that overexpression of ANKS1B did not alter drug intake over the 17-day acquisition period compared to the control virus group . The right panel shows that knockdown of ANKS1B also did not affect drug intake (Two-way RM ANOVA, p > 0.05). n = 7 per group. Data are presented as mean ± SEM.


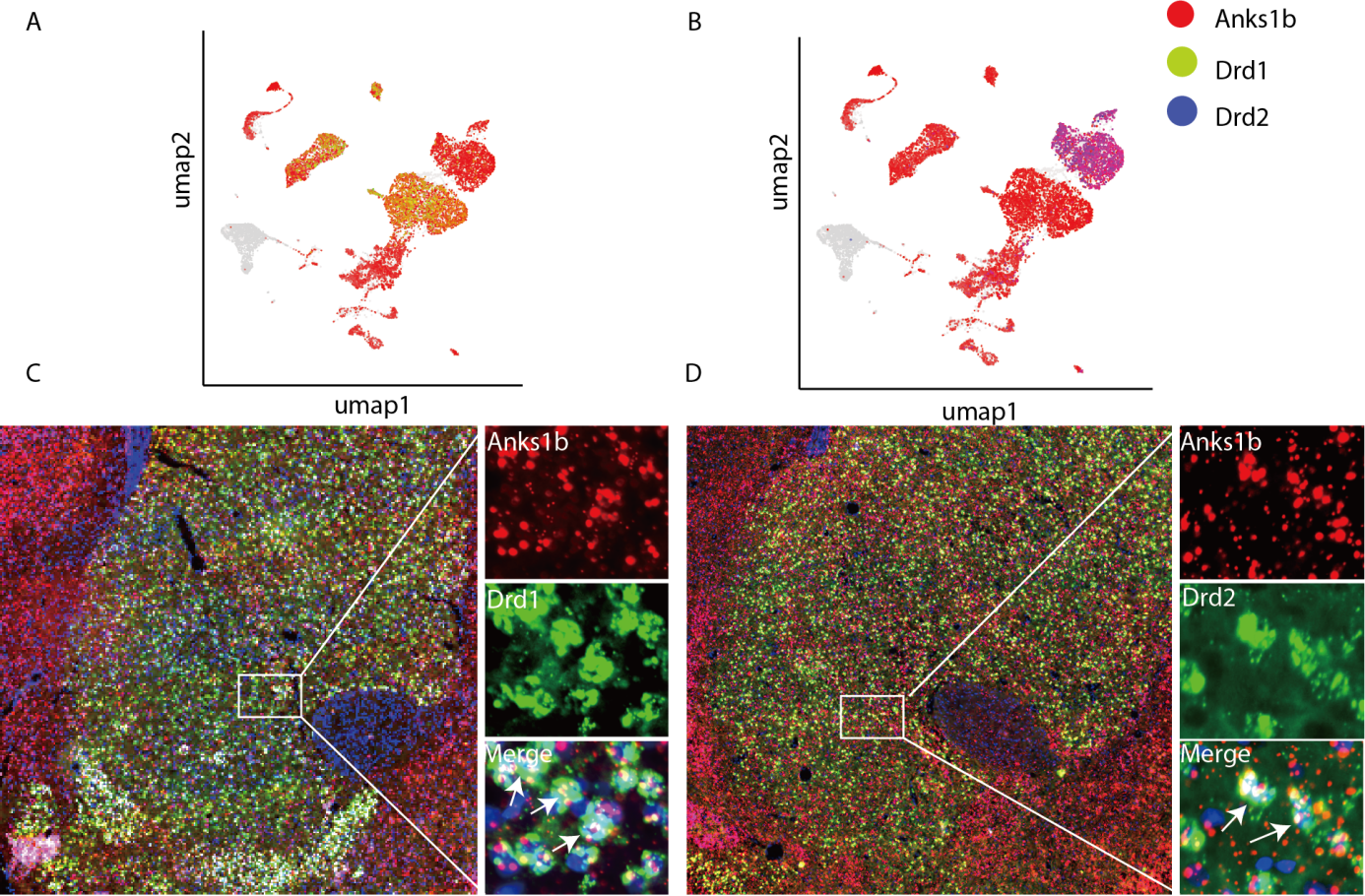


**Figure S5. *Anks1b* is expressed in both D1- and D2-receptor-expressing medium spiny neurons (MSNs) in the NAc.** **(A–B)** UMAP (Uniform Manifold Approximation and Projection) plots of single-cell RNA-sequencing data from the NAc. A) shows the expression distribution of the *Anks1b* gene (red indicates high expression). B) shows the expression distribution of dopamine D1 receptor (*Drd1*, green) and D2 receptor (*Drd2*, blue). Comparison of the plots indicates that *Anks1b* is expressed in both D1-MSNs and D2-MSNs. **(C–D)** Fluorescent in situ hybridization (FISH) images confirming the co-localization of *Anks1b* mRNA (red) with *Drd1/Drd2* mRNA (green) in NAc neurons (yellow signal in Merge panels, indicated by arrows).


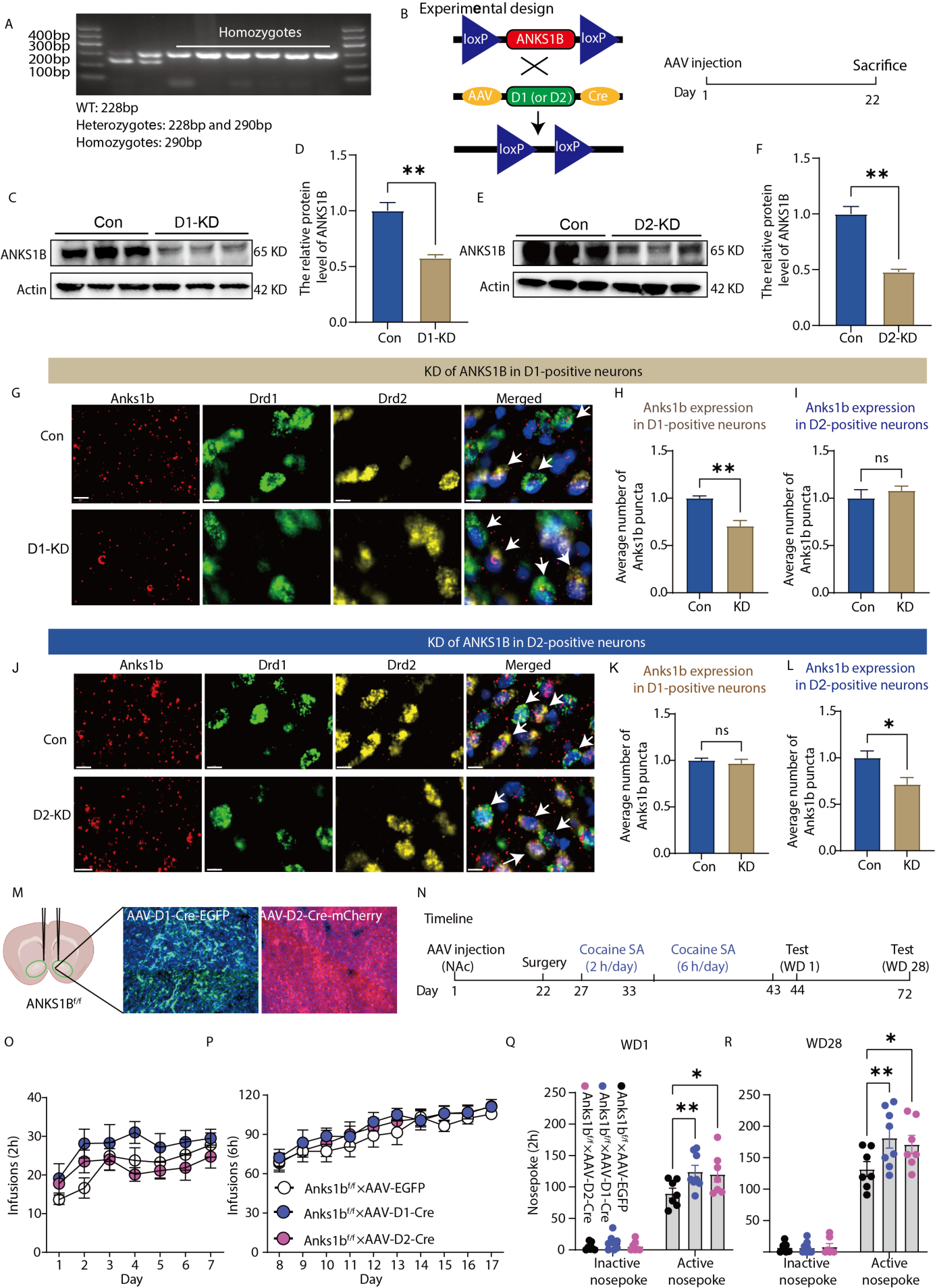


**Figure S6. Cell-type–specific deletion of ANKS1B in NAc D1- or D2-MSNs regulates cocaine seeking. (A)** PCR genotyping of Anks1b^f/f^ rats showing WT (228 bp), heterozygous (228/290 bp), and homozygous (290 bp) bands. **(B)** Schematic workflow of the experimental design. AAV-D1-Cre or AAV-D2-Cre was injected into the NAc of Anks1b^f/f^ rat to induce site-specific recombination of loxP sites. Tissues were harvested for analysis 21 days post-injection. **(C-D)** Western blot validation and quantification of ANKS1B protein levels in the NAc after D1-KD (unpaired t-test, t_4_ = 5.30, P = 0.01, n = 3 per group). **(E–F)** Western blot validation and quantification after D2-KD (unpaired t-test, t_4_ = 7.37, P < 0.01, n = 3 per group). Representative RNAscope images of Anks1b mRNA (red) with Drd1 (green) and Drd2 (yellow) labeling in the NAc. In Anks1b ^f/f^ × AAV-D1-Cre animals (D1-MSN-specific ANKS1B KD), Anks1b puncta are selectively reduced in Drd1-positive neurons but not in Drd2-positive neurons. Arrows indicate colocalized puncta. All scale bars, 10 µm. **(G)** Representative RNAscope images of Anks1b mRNA (red) with Drd1 (green) and Drd2 (yellow) labeling in the NAc. In Anks1b^f/f^ × AAV-D1-Cre animals (D1-MSN-specific ANKS1B KD), Anks1b puncta are selectively reduced in Drd1-positive neurons but not in Drd2-positive neurons. Arrows indicate colocalized puncta. All scale bars, 10 µm. H, I) Quantification of Anks1b puncta in D1- and D2-MSNs following D1-KD. Anks1b signal is significantly reduced in D1-MSNs but unchanged in D2-MSNs (unpaired t-test, t_6_ = 4.62, P < 0.01; t_6_ = 0.80, P = 0.46; n = 4 per group). **(H–I)** Quantification of Anks1b puncta in D1- and D2-MSNs following D1-KD. Anks1b signal is significantly reduced in D1-MSNs but unchanged in D2-MSNs (unpaired t-test, t_6_ = 4.62, P < 0.01; t_6_ = 0.80, P = 0.46; n = 4 per group). **(J)** Representative RNAscope images of Anks1b mRNA (red) with Drd1 (green) and Drd2 (yellow) labeling in the NAc. In Anks1b^f/f^ × AAV-D2-Cre animals (D2-MSN-specific ANKS1B KD), Anks1b puncta are selectively reduced in Drd2-positive neurons but not in Drd1-positive neurons. Arrows indicate colocalized puncta. All scale bars, 10 µm. **(K–L)** Quantification of Anks1b puncta following D2-KD. Anks1b expression is selectively reduced in D2-MSNs, with no effect in D1-MSNs (unpaired t-test, t6 = 0.63, P = 0.55; t6 = 2.74 P = 0.03, n = 4 per group). **(M)** Representative images confirming successful infection of NAc neurons. Left: schematic illustration of injection sites in the NAc. Middle: AAV-D1-Cre-EGFP expression (green) in NAc. Right: AAV-D2-Cre-mCherry expression (red) in NAc. **(N)** Experimental timeline. After viral injection and recovery, rats underwent short-access cocaine self-administration (SA; 2 h/day) for 7 days, followed by long-access cocaine SA (6 h/day) for 10 days. Drug-seeking behavior was tested on WD1 and WD28 under extinction conditions. **(O)** Cocaine intake during 2 h/day SA sessions. Deletion of ANKS1B in either D1- or D2-MSNs did not alter cocaine intake relative to controls. **(P)** Cocaine intake during 6 h/day SA sessions (long access). All groups exhibited escalation of intake across training days, with no significant differences between groups. **(Q)** Cocaine seeking at WD1. A two-way RM analysis revealed a significant main effect of genotype (F_2, 19_ = 5.48, P = 0.01), with no significant genotype × lever interaction (F_2, 19_ = 2.27, P = 0.12). Post hoc Dunnett’s multiple-comparisons test showed that both Anks1b^f/f^ × D1-Cre and Anks1b^f/f^ × D2-Cre groups exhibited significantly increased active nosepokes compared with controls (P = 0.01 and P = 0.02, respectively), whereas no differences were observed in inactive nosepokes. **(R)** Cocaine seeking at WD28. A two-way RM analysis revealed a significant lever × genotype interaction (F_2, 19_ = 4.58, P = 0.02). Post hoc Dunnett’s multiple-comparisons test showed that both Anks1b^f/f^ × D1-Cre and Anks1b^f/f^ ×D2-Cre groups exhibited significantly increased active nosepokes compared with controls (p < 0.01 and p = 0.03, respectively), whereas no differences were observed in inactive nosepokes. n = 7–8 per group. Data are presented as mean ± SEM. P < 0.05 was considered statistically significant. Statistical analysis was performed using two-way ANOVA followed by Dunnett’s post hoc multiple-comparisons test.


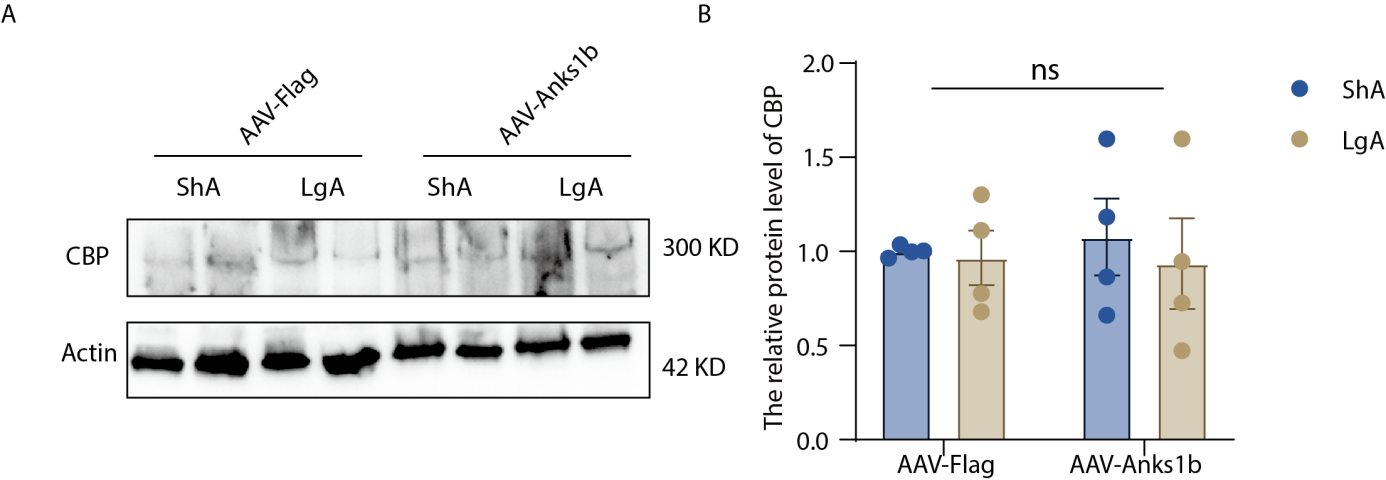


**Figure S7. ANKS1B overexpression does not alter total CBP protein levels in the NAc following cocaine self-administration. (A–B)**.Representative western blot images and quantitative analyses indicate that neither extended cocaine self-administration nor ANKS1B overexpression alters CBP protein levels in the nucleus accumbens. (two-way ANOVA, genotype × access interaction: F_1, 12_ = 0.09, P = 0.76; n = 4 per group). Data are presented as mean ± SEM. P < 0.05 was considered statistically significant. Statistical significance was determined by two-way ANOVA followed by Tukey’s post hoc test.


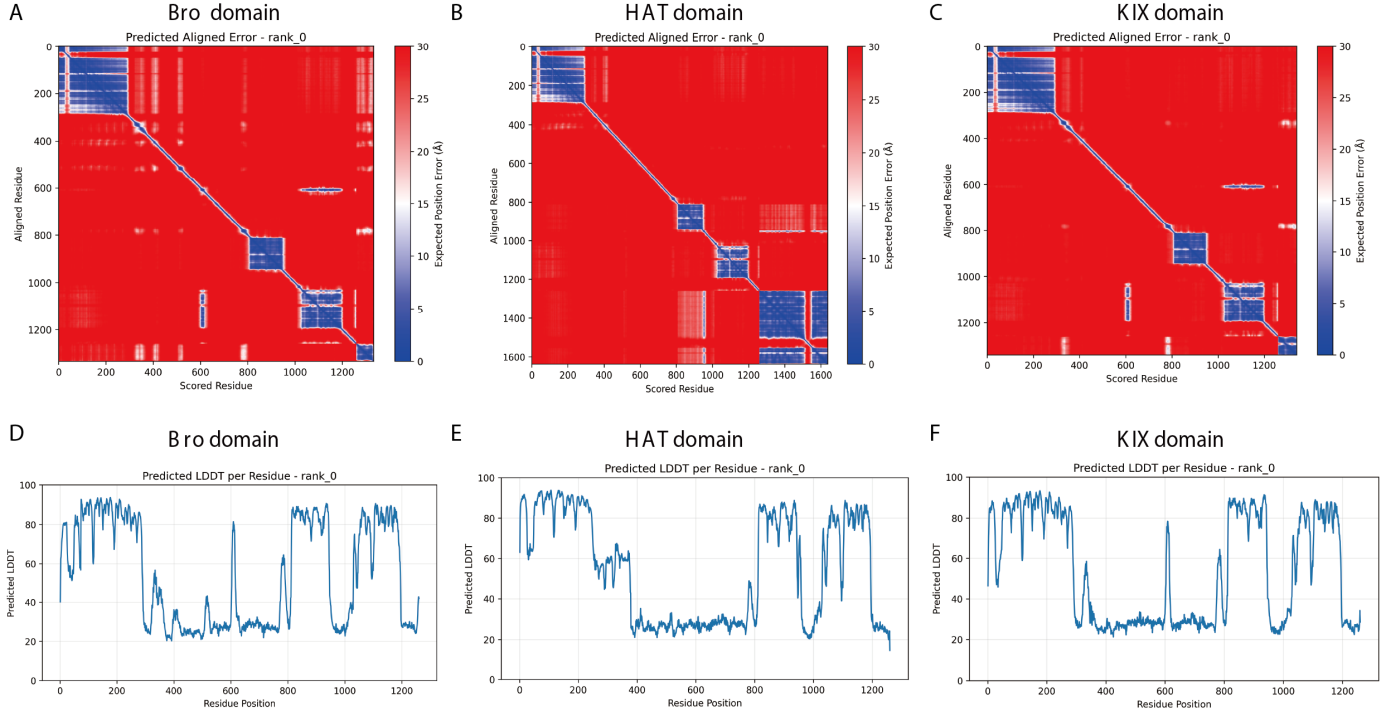


**Figure S8. Structural prediction confidence of ANKS1B-CBP domain interactions based on AlphaFold analysis. (A–C)** Predicted aligned error (PAE) plots for ANKS1B in complex with CBP domains, including the Bro domain (A), HAT domain (B), and KIX domain (C). Lower PAE values (blue) indicate higher confidence in the relative positioning of residues, whereas higher values (red) indicate lower confidence. **(D–F)** Predicted local distance difference test (pLDDT) scores per residue for the corresponding complexes shown in (A–C), including the Bro domain (D), HAT domain (E), and KIX domain (F). Higher pLDDT scores indicate greater confidence in local structural predictions.


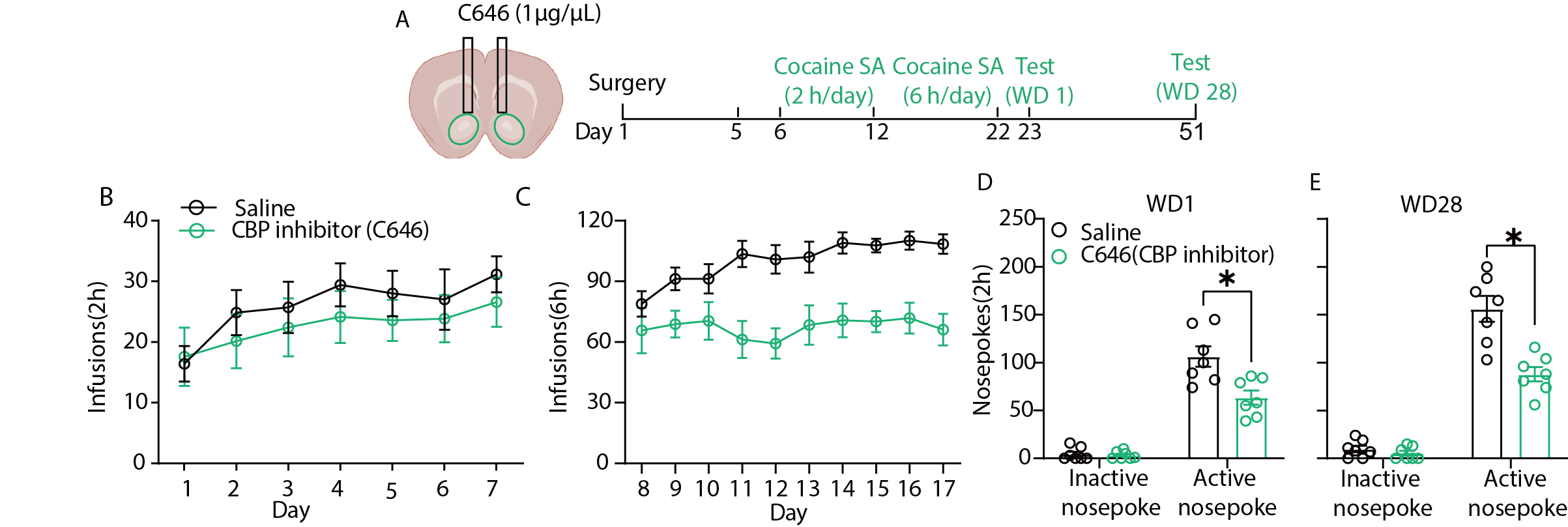


**Figure S9. Pharmacological inhibition of CBP in the NAc decreases escalated cocaine intake and seeking.** **(A)** Schematic of the experimental design, involving direct infusion of the CBP inhibitor C646 into the bilateral NAc. **(B)** C646 had no significant effect on cocaine intake during the short-access (2h/day) phase. **(C)** During the long-access (6h/day) phase, C646 significantly decreased the escalation of cocaine intake. **(D, E)** C646 significantly decreased cue-induced active nosepokes on both WD1 and WD28 (WD1: two-way RM ANOVA, genotype × nosepoke interactions: F_1, 12_ = 9.12, P = 0.01; post hoc, P < 0.01; WD28: two-way RM ANOVA, treatment × nosepoke interactions: F_1, 12_ = 17.81, P < 0.01; post hoc, P < 0.01; n = 7 per group). Data are presented as mean ± SEM. P < 0.05 was considered statistically significant. Statistical significance was determined by two-way repeated-measures (RM) ANOVA followed by Bonferroni's multiple comparisons test.


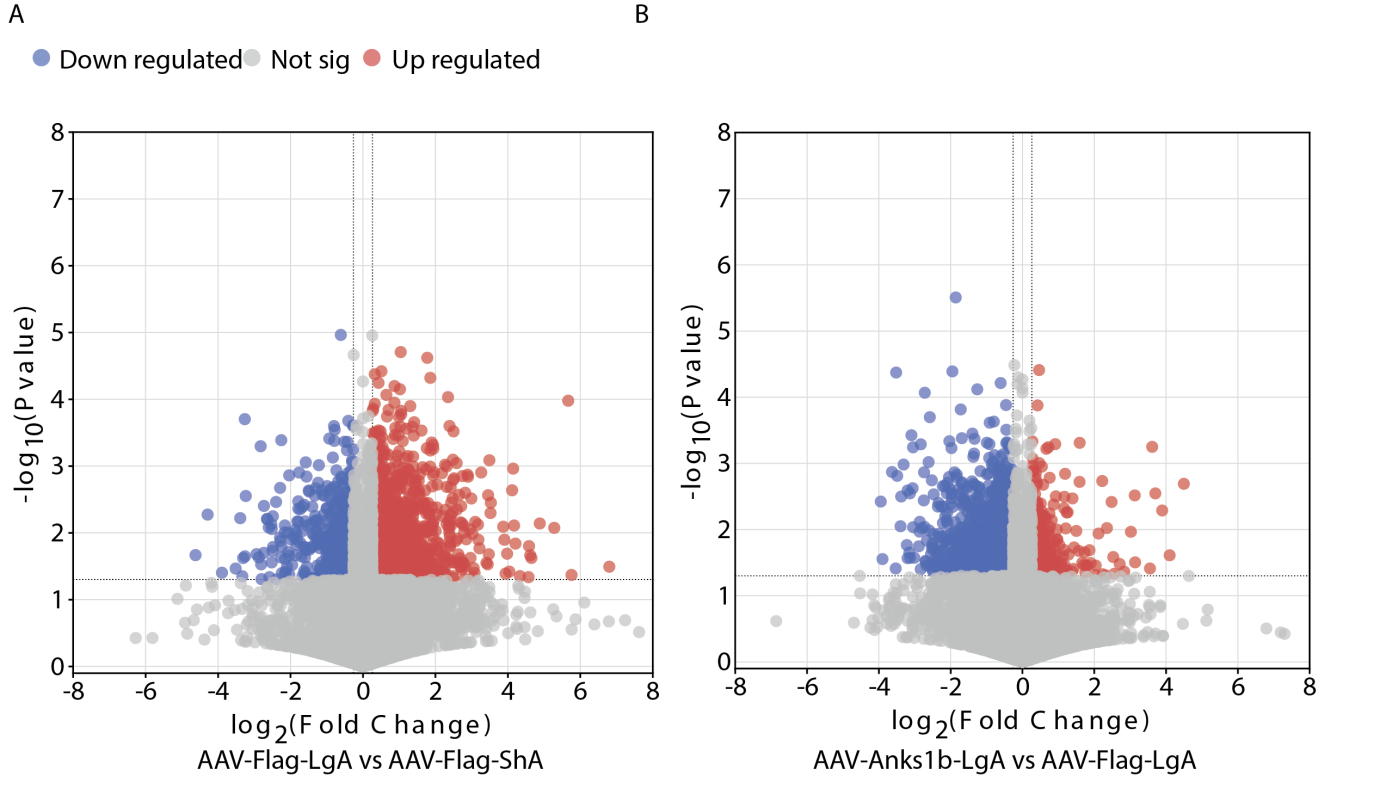


**Figure S10. Transcriptomic analysis of gene expression changes in the Nucleus Accumbens (NAc).** **(A)** Volcano plot illustrating differentially expressed genes in the NAc between the long-access (LgA) and short-access (ShA) cocaine self-administration groups. Red dots indicate significantly upregulated genes, and blue dots indicate significantly downregulated genes in the LgA group compared to the ShA group. **(B)** Volcano plot illustrating differentially expressed genes between the ANKS1B overexpression LgA group (OE-LgA) and the LgA control group. Genes downregulated by LgA are now upregulated (red dots) and genes upregulated by LgA are now downregulated (blue dots).


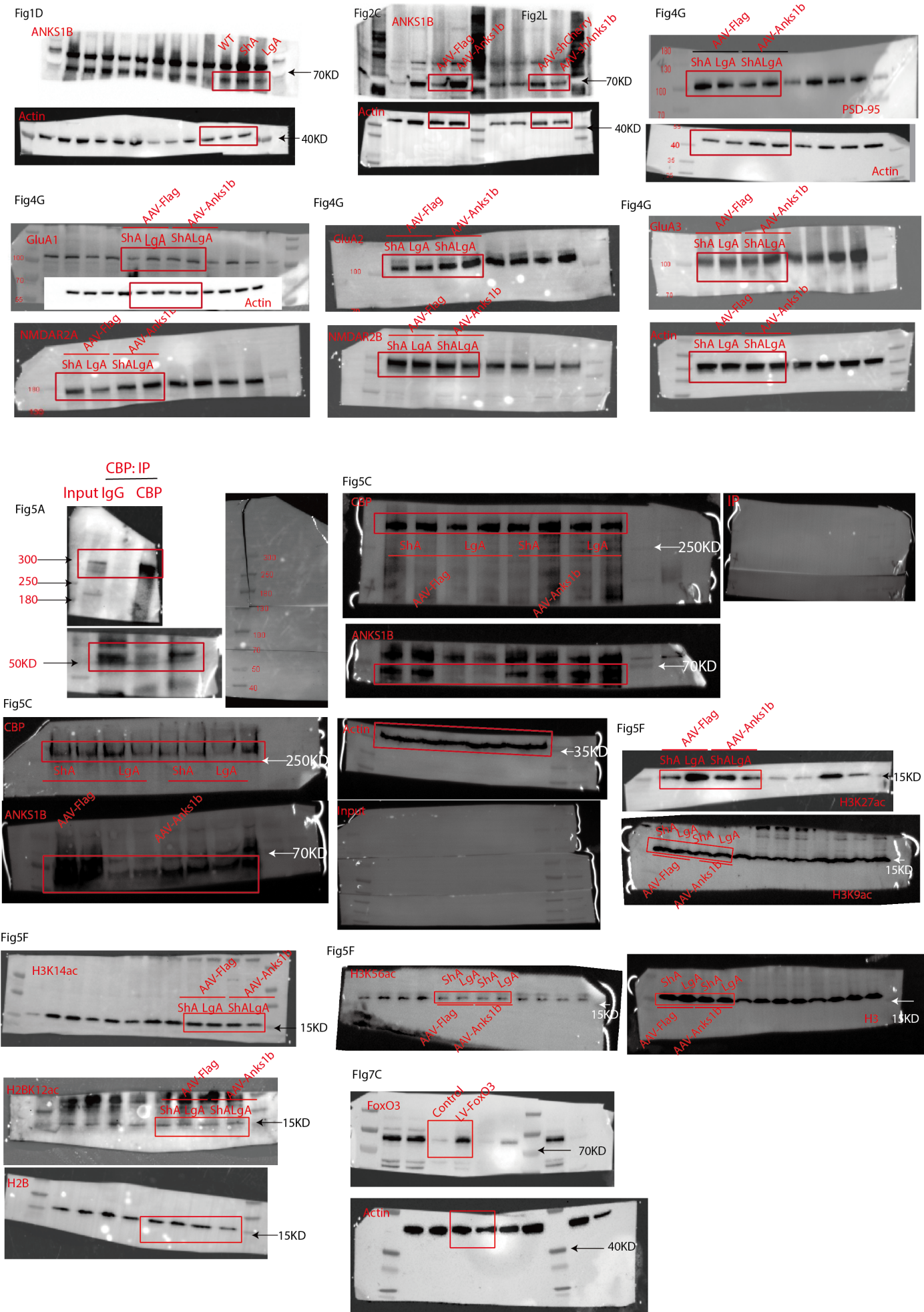


**Fig S11.** Unedited blot and gel images

**Table S1. The sequences of used primers for RT-qPCR.**

| Gene | Forward primer | Reverse primer |
| --- | --- | --- |
| *FoxO1* | AAATGGGAGATTGGGCCTGG | TGAATGCCAGTCCACCATCC |
| *FoxO3* | CAGACCCTCAAACTGACCGAA | GCTGCTAACAGTCTCTGCTGG |
| *FoxO6* | ACTCCATCCGGCACAACCT | TTGCCAGTCCCCTCATTCTG |

**Table S2. The sequences of used primers for CHIP-qPCR.**

| Gene | Forward primer | Reverse primer |
| --- | --- | --- |
| *FoxO1* | CAAAACAAACCCCACCGACT | GCGGCGACCATACTTTTGTT |
| *FoxO3* | AACCGGAAAAGCGTAGCTCA | GGTATAGGGTTGTCGCGGAG |
| *FoxO6* | GCAAAGACACCAACAGGCAG | GTCTGTCCAAGTACACCGCA |

**TableS3. Predicted confidence metrics for ANKS1B–CBP domain interaction models generated by AlphaFold3.**

|  | KIX | HAT | BRO |
| --- | --- | --- | --- |
| pTM | 0.33 | 0.34 | 0.31 |
| ipTM | 0.49 | 0.70 | 0.46 |
| Ranking Score | 0.66 | 0.82 | 0.65 |
